# Willingness to practice medicine and related influential factors among medical undergraduates during COVID-19: a cross-sectional study

**DOI:** 10.1186/s12909-023-04418-7

**Published:** 2023-06-08

**Authors:** Shuang Yu, Fengjuan Zou, Qian Wang, Kai Zhou, Ronghua Jian, Yingying Jin, Yijun Hu, Sui Zhu

**Affiliations:** 1 Department of Medical Education, Nanfang Hospital, Southern Medical University, Guangzhou, 510515 China; 2 Department of Epidemiology, School of Medicine, Jinan University, No.601 Huangpu Road West, Guangzhou, 510632 China; 3 Infection Control Department, Guangzhou First People’s Hospital, School of Medicine, South China university of Technology, Guangzhou, 510050 China; 4 Faculty of Medicine, International School, Jinan University, Guangzhou, 510632 China

**Keywords:** Medical undergraduates, Willingness to practice medicine, Influential factors

## Abstract

**Background:**

As the medical undergraduates constitute the future workforce in China, their career preferences hold a significant bearing on the quality of healthcare services, particularly in light of the ongoing COVID-19 pandemic. We aim to understand the current state of the willingness to practice medicine among medical undergraduates and to analyze the related influential factors.

**Methods:**

During the COVID-19 epidemic, we conducted a cross-sectional survey via an online platform from February 15, 2022, to May 31, 2022, to collect participants’ demographic information, psychology, and factors influencing their career choices. The general self-efficacy scale (GSES) was used to evaluate medical students’ perceptions of their self-efficacy. Futhermore, we conducted multivariate logistic regression analyses to explore the influencing factors of medical undergraduates’ willingness to pursure a caree in medicine.

**Results:**

A total of 2348 valid questionnaires were included, and 1573 (66.99%) were willing to practice medicine for medical undergraduates after graduation. The mean GESE scores in the willingness group (2.87 ± 0.54) were significantly higher than those of the unwillingness group (2.73 ± 0.49). The multiple logistic regression showed that several factors were positively associated with willingness to practice medicine as a career, including students’ GSES score (OR = 1.87), current major, household income, personal ideals (OR = 1.97), family support (OR = 1.44), high income (OR = 1.77), and social respect (OR = 2.19). Compared with those who were very afraid of COVID-19, students who did not express any fear towards the COVID-19 pandemic had a higher preference for choosing the medical profession as a career. Conversely, students thinking of high tension in the doctor-patient relationship, heavy workload, and long training were less likely to choose medical work after graduation.

**Conclusions:**

The study highlights a noteworthy prevalence of medical undergraduates who expressed their willingness to pursue medicine as a career post-graduation. Several factors, including but not limited to current major, household income, psychological factors, personal preferences, and career needs or preferences, were significantly associated with this willingness. Moreover, the impact of the COVID-19 pandemic on medical students’ career choices cannot be overlooked.

**Supplementary Information:**

The online version contains supplementary material available at 10.1186/s12909-023-04418-7.

## Introduction

With rapid aging and changes in disease patterns, the number of registered doctors and nurses per 1000 residents has recently increased in China [1]. According to the Global Health Workforce Statistics database, there is still a gap in achieving the Healthy China 2030 Plan (including 3 registered doctors and 4.7 registered nurses per 1000 residents by 2030) [2]. In 2019, for every 1000 people in the population, there were only 2.23 medical doctors (including assistant doctors) in China [3]. Although there is no shortage of medical students, dropping out of medical school is problematic. A study indicated that dropping out of medical school ranged between 2.7% and 20.1% [4], and between 2004 and 2008, only 20–40% of medical graduates were employed as health workers in China [5]. A recent study suggests that approximately 14% of new medical school graduates have other career choices [6]. Medical undergraduates represent the future workforce of the medical industry, and their job preferences can directly impact the development of the industry and the quality of healthcare services in the future. Thus, understanding the willingness of medical undergraduates to practice medicine is crucial in the modern healthcare industry in China.

The factors that influence medical students’ career choices are numerous and complex. Some of the most common factors include students’ characteristics, family influence, financial considerations, academic performance, healthcare workplace violence, and working hours [7–9]. In China, the number of patient visits per doctor increased by 135%, and the number of inpatient admissions per doctor rose by 184% between 1998 and 2016, which indicated that doctors’ workload had markedly increased [10]. Work-related problems of doctors are growing public concerns [11]. For example, over 32% of doctors work more than 60 h per week in China, violating the legal limits of China for 44 h of work per week [10], and there is a serious imbalance between the low income of doctors and the heavy workload [12]. Moreover, the frequent occurrence of doctor-patient conflicts and negative reports in the media have increased the public’s negative perception of the healthcare industry and medical staff, resulting in a crisis of trust [13], which has also led to an increase in the occupational risk of medical workers due to incidents of violence against doctors [14].

Since the outbreak of the COVID-19 pandemic, the global community has witnessed remarkable and unparalleled displays of solidarity and resilience from healthcare workers. Various relevant environmental changes resulting from the epidemic inevitably affect medical students’ thoughts when planning their careers. China implemented a “zero-tolerance” policy, resulting in the closure of schools and factories, and all the pressure was placed on front-line medical workers (including doctors, community healthcare workers, and nurses), who became even more essential and visible, fighting against the virus and risking their health and safety to provide care and support to those affected [15]. Meanwhile, the dedication of healthcare professionals has earned them the admiration of society as a whole, leading to a notable enhancement in the rapport between doctors and patients [16]. In addition, some medical students were also at the forefront of the fight against the virus, playing a crucial role in treating patients and preventing the further spread of the disease. This experience has had a significant impact on the career choices of medical students, with the dropout intention rate among Chinese undergraduate medical students decreasing from 13.7 to 6.8% [6]. However, the main time for medical students to establish their professional direction is internship and probation, so the failure to complete the internship plan during the COVID-19 epidemic will have a huge impact on their career choice.

Previous studies have mostly focused on influencing factors about the specialty or subspecialty choice for medical students [17–19]. An imbalance in the supply of doctors in different specialties and subspecialties is vital to the healthcare industry. However, most medical undergraduates in China do not specialize in a particular field during their undergraduate studies. They choose specialties and subspecialties only after their graduate studies. Henceforth, understanding whether medical graduates are willing to work in the healthcare industry is particularly important to remedy uneven distribution across specialties. In addition, the various objective environmental and educational changes caused by the COVID-19 pandemic will inevitably lead to new considerations or changes in medical students’ career choices. The impact of these changes on the psychological well-being of medical students also partly determines whether they choose to pursue further medical education, and to some extent, may also lead to changes in their career choices [20].

At present, there exists a conspicuous insufficiency of research on the aforementioned topic concerning medical undergraduates in China. By understanding the career choices of medical undergraduates, we can learn about their views on the challenges and problems in the healthcare industry, provide better welfare and career development opportunities to attract and retain excellent medical students, and help to promote the development of the healthcare industry, especially in promoting innovation and development of medical technologies and services. Therefore, we conducted a cross-sectional study to (1) understand the current state of willingness to practice medicine among medical undergraduates; and (2) identify the factors including demographic characteristics, psychological factors, and motivations for career choice that influence willingness to practice medicine. The results of this study can potentially bridge this gap in knowledge and can help policy-makers to develop more reasonable and targeted recruitment and training plans.

## Materials and methods

### Study participants and procedure

A cross-sectional study was carried out among medical undergraduates in several of China’s higher medical schools from February 15, 2022, to May 31, 2022. A structured online questionnaire was distributed through the WenJuanXing internet-based survey platform (www.wjx.cn) and WeChat. All participants provided informed consent before answering the questionnaire, and the questionnaire required approximately 15 min to be completed. Those respondents who answered all inquiries were able to submit the questionnaire. The confidentiality and anonymity of the participants’ information were ensured throughout the study.

### Demographic characteristics

The demographic characteristics of the students included in the study were age, gender, race, current major, grade, family living area, father’s or mother’s education, household income, and family members or relatives in healthcare. The participants’ grade levels were categorized into three levels: freshman, sophomore and junior were considered the low grade, while senior and fifth grade were classified as the high grade.

### The general self-efficacy scale (GSES)

The GSES has been widely used globally to assess medical staff’s self-efficacy perceptions [21]. We used the Chinese version of the GSES, which consists of 10 items, and each item is rated on a 4-point Likert scale from 1 (“strongly agree”) to 4 (“strongly disagree”), resulting in a total score ranging from 10 to 40, with higher scores indicating greater self-efficacy [22]. The internal consistency for using the GSES had good internal reliability in our study ($a$ = 0.817).

### Career choice and motivation factors

The outcome of this study was the career decision of medical undergraduates with regard to practicing medicine upon completion of their studies, and all the participants were asked whether they had thought about it. Those students who answered affirmatively were categorized as the “willingness” group, while the others were regarded as the “unwillingness” group.

To investigate the underlying motivations for career choice, students were required to select the reason(s) for their career choice. The options included reasons for choosing medicine by yourself or your parents’, ‘family expectations’, ‘family support for medical practice’, ‘helping family members in future’, ‘the job is stable’, ‘high income’, ‘high social respect’, ‘high employment rate’, ‘poor doctor-patient relationships’, ‘heavy workload’, ‘long study period,’ ‘high risk of infection in healthcare’, and ‘whether you are afraid of COVID-19’. The degree of fear of COVID-19 used a 4-point Likert scale: “very afraid”, “afraid”, “not afraid” and “not afraid at all”. Before the formal survey, a small preliminary survey was conducted among medical undergraduates at a university in Guangdong Province to ensure that the questionnaire was easy to understand and complete.

### Statistical analysis

The age and GSES were presented as the means ± standard deviations (SD) for normally distributed variables using the Shapiro-Wilk normality test. The GSES score was divided by 10 with the final points, and Cronbach’s $a$ coefficient was calculated as a measure of internal consistency for the GSES. Student’s *t* test was used to compare the continuous variables between the willingness and unwillingness groups. Categorical data were expressed as counts and percentages, and Chi-square tests and Wilcoxon rank-sum tests were used to explore the difference between the two groups. Multivariate logistic regression was used to assess the association of basic characteristics, psychological factors, and motivations for career choice. All statistical analyses were completed using R software (version 4.2.2), and the statistically significant test level was set at *P* < 0.05.

## Results

### Current state of willingness to practice medicine

A total of 2369 medical undergraduates participated in this study. After checking the time of questionnaire completion and the options before and after the questions: 21 invalid questionnaires were excluded from the analysis. Thus, a total of 2348 valid questionnaires were included with an average age of 21.0 ± 1.3 years old. Of the 2348 medical undergraduates, 1573 (66.99%) were willing to engage in the medical profession, while 775 (33.01%) were unwilling to engage in the medical profession after graduation. Approximately 55.83% of medical undergraduates who participated in the study were female, 81.35% identified as Han nationality, and 76.19% reported that their families lived on the mainland.

### Willingness to practice medicine among demographic characteristics

The mean ages in the unwillingness and willingness groups were 20.95 ± 1.41 and 21.02 ± 1.24 years old, respectively, without a significant difference (*P* = 0.238). Notably, there were significant differences in the proportions of current major, undergraduate years, and family members or relatives in healthcare between the willingness and unwillingness groups (*P* < 0.05, Table 1). In the willingness group, the proportions of individuals involved in the field of clinical medicine (33.95% vs. 25.16%) and traditional Chinese medicine (16.02% vs. 11.35%) were comparatively higher in comparison to the unwillingness group. Students in high grades had a higher proportion of choosing the medical profession as a career (36.24% vs. 29.03%). The percentage of family members or relatives working in healthcare was 75.14% in the willingness group, which was higher than the corresponding figure in the unwillingness group (69.94%) after graduation (*P* = 0.007). No statistically significant differences were observed in the proportions of other demographic characteristics between two groups (*P* > 0.05).

**Table 1:** The comparisons of the distribution of willingness to practice medicine among various demographic characteristics of medical undergraduates in China

Variables	Unwillingness (*n* = 775)	Willingness (*n* = 1573)	*P*
Age, year (Mean ± SD)	20.95 ± 1.41	21.02 ± 1.24	0.238
Gender (*n* [%])			0.840
Male	340 (43.87)	697 (44.31)	
Female	435 (56.13)	876 (55.69)	
Race (n [%])			0.342
Han nationality	622 (80.26)	1288 (81.88)	
Others	153 (19.74)	285 (18.12)	
Family living area (n [%])			0.878
Mainland	589 (76.00)	1200 (76.29)	
Others	186 (24.00)	373 (23.71)	
Current major (n [%])			< 0.001
Pharmacology	133 (17.16)	140 (8.90)	
Clinical medicine	195 (25.16)	534 (33.95)	
Dental medicine	191 (24.65)	365 (23.20)	
Nurse	168 (21.68)	282 (17.93)	
Traditional Chinese medicine	88 (11.35)	252 (16.02)	
Undergraduate years (n [%])			< 0.001^*^
Freshman	176 (22.71)	299 (19.00)	
Low grade	374 (48.26)	704 (44.76)	
High grade	225 (29.03)	570 (36.24)	
Father’s or mother’s education (n [%])			0.152^*^
Below primary school	137 (17.68)	322 (20.47)	
Junior school	210 (27.09)	441 (28.04)	
High school	221 (28.52)	395 (25.11)	
University and above	207 (26.71)	415 (26.38)	
Household income (thousands per year) (n [%])			0.085^*^
< 50 RMB	206 (26.58)	355 (22.57)	
50 ~ 99 RMB	218 (28.13)	591 (37.57)	
100 ~ 200 RMB	176 (22.71)	398 (25.30)	
≥201 RMB	175 (22.58)	229 (14.56)	
Family members or relatives in healthcare (n [%])			0.007
No	233 (30.06)	391 (24.86)	
Yes	542 (69.94)	1182 (75.14)	

^*^ Nonparametric test was used for ordinal categorical data

### Comparison of score for GSES

The mean GSES score in all participants was 2.83 ± 0.53, which was at the medium level. There was a significant difference in GESE scores between the two groups, with higher scores in the willingness to practice medicine (2.87 ± 0.54) than in the unwillingness group (2.73 ± 0.49).

### Motivation for students’ willingness to practice medicine

In order to further investigate the underlying motivations for the choice in the medical profession, the comparison analysis for medical undergraduates’ willingness was assessed, as shown in Table 2. Students motivated by personal ideals (59% vs.40.26%), family support (64.65% vs. 57.03%), high income (67.01% vs. 53.03%), and social respect (80.99% vs. 66.97%) were significantly more likely to think of the selection of a medical profession in the future compared with other students (*P* < 0.001). In contrast, unwillingness to practice medicine were higher among medical undergraduates with high tension in the doctor-patient relationship, heavy workload, and long training (*P* < 0.001). In addition, students who were very afraid and afraid of COVID-19 were unwilling to choose to engage in the medical profession (*P* < 0.001).

**Table 2:** Comparison analysis for underlying motivation factors in medical undergraduates’ willingness to practice medicine

Variables	Unwillingness (*n* = 775)	Willingness (*n* = 1573)	*P*
Personal ideals (n [%])			< 0.001
No	463 (59.74)	645 (41.00)	
Yes	312 (40.26)	928 (59.00)	
Family expectations (n [%])			0.514
No	387 (49.94)	808 (51.37)	
Yes	388 (50.06)	765 (48.63)	
Family support (n [%])			< 0.001
No	333 (42.97)	556 (35.35)	
Yes	442 (57.03)	1017 (64.65)	
Helping family members (n [%])			0.137
No	201 (25.94)	454 (28.86)	
Yes	574 (74.06)	1119 (71.14)	
Job Stability (n [%])			0.528
No	124 (16.00)	236 (15.00)	
Yes	651 (84.00)	1337 (85.00)	
High income (n [%])			< 0.001
No	364 (46.97)	519 (32.99)	
Yes	411 (53.03)	1054 (67.01)	
Social respect (n [%])			< 0.001
No	256 (33.03)	299 (19.01)	
Yes	519 (66.97)	1274 (80.99)	
High employment rate (n [%])			0.214
No	244 (31.48)	456 (28.99)	
Yes	531 (68.52)	1117 (71.01)	
Tension in doctor-patient relationship (n [%])			< 0.001
No	263 (33.94)	724 (46.03)	
Yes	512 (66.06)	849 (53.97)	
Heavy workload (n [%])			< 0.001
No	325 (41.94)	897 (57.02)	
Yes	450 (58.06)	676 (42.98)	
Long training (n [%])			< 0.001
No	240 (30.97)	630 (40.05)	
Yes	535 (69.03)	943 (59.95)	
High risk of infection (n [%])			0.391
No	212 (27.35)	457 (29.05)	
Yes	563 (72.65)	1116 (70.95)	
Afraid of COVID-19 degree (n [%])			< 0.001
Very afraid	147 (18.97)	236 (15.00)	
Afraid	256 (33.03)	440 (27.97)	
Not afraid	178 (22.97)	346 (22.00)	
Not afraid at all	194 (25.03)	551 (35.03)	

### Multivariate factors influencing willingness to practice medicine

**Fig. 1:**
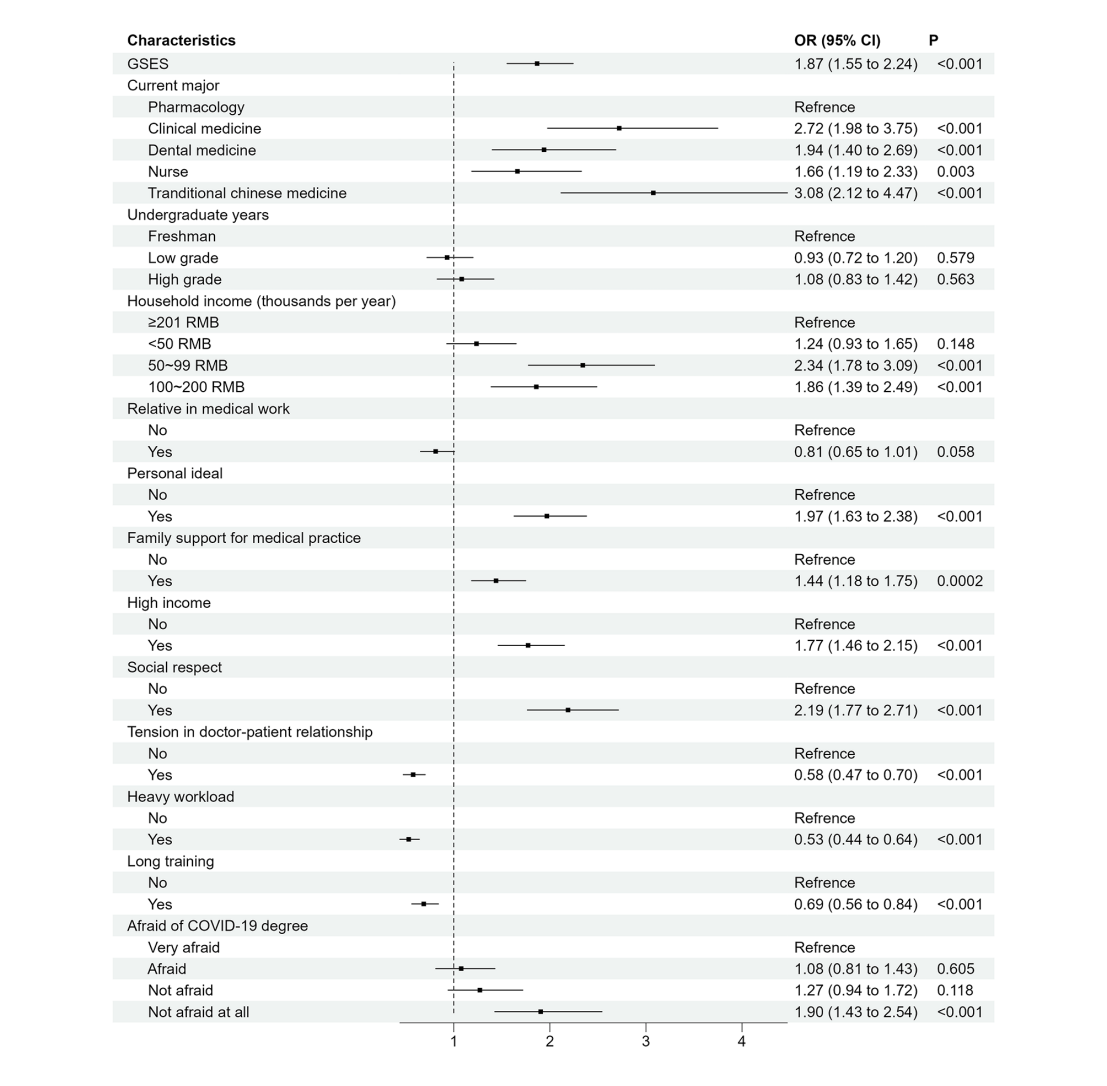
Multivariable regression analysis of influential factors for willingness to practice medicine

The dependent variable was whether medical undergraduates were willing to engage in the medical profession after graduation, and the independent variables were selected based on statistically significant rates of willingness to practice medicine, as illustrated in Tables 1 and 2. For the corresponding variable codes for both dependent and independent variables, see supplementary Table S1. Figure 1 shows a multivariate logistic regression result of all dimensions of demographic characteristics, psychological factors, and motivations for career choice.

Several factors were positively associated with willingness to practice medicine as a career, including students’ GSES score (OR = 1.87, 95% CI:1.55 to 2.24), personal ideal (OR = 1.97, 95% CI:1.63 to 2.38), family support (OR = 1.44, 95% CI:1.18 to 1.75), high income (OR = 1.77, 95% CI:1.46 to 2.15), and social respect (OR = 2.19, 95% CI:1.77 to 2.71). Compared with students majoring in pharmacology, students majoring in clinical medicine (OR = 2.72, 95% CI: 1.98 to 3.75), dentistry (OR = 1.94, 95% CI: 1.40 to 2.69), traditional Chinese medicine (OR = 3.08, 95% CI: 2.12 to 4.47) and nursing (OR = 1.66, 95% CI: 1.19 to 2.33) were more prone to willingness to practice medicine as a career. Additionally, students with household income ranging from 50 ~ 200 RMB exhibited a higher preferences for choosing the medical profession (OR = 2.34 and OR = 1.86, respectively) in comparison to the group with a household income of ≥ 201 RBM. Meanwhile, a higher preference for choosing the medical profession as a career was found in the students who were not afraid at all (OR = 1.90, 95% CI:1.43 to 2.54, *P* < 0.001). Students thinking of high tension in the doctor-patient relationship (OR = 0.58, 95% CI:0.47 to 0.70), heavy workload (OR = 0.53, 95% CI:0.44 to 0.64), and long training (OR = 0.69, 95% CI:0.56 to 0.84) were less likely to choose medical work after graduation. Other variables were not correlated with career choice, as shown in Fig. 1.

## Discussion

The present study employed a cross-sectional survey to investigate the attributes characteristics of willingness to practice medicine, as well as the correlated factors that influence career choice among undergraduate students at medical universities in China. Understanding the job preferences of medical undergraduates is critical in achieving high-quality healthcare services and the sustainable development of the healthcare industry in China. In this study, we found that 66.99% of medical undergraduates were willing to engage in the medical profession after graduation, and several factors were positively associated with willingness to practice medicine, including increasing students’ GSES score, current major, personal ideal to choose medicine, and family support. Regarding the motivations for career choice, the high income and social respect were critical in promoting the job preferences of medical undergraduates. At the same time, tension in the doctor-patient relationship, heavy workload, and long training were negatively related to the willingness for job preferences of medical undergraduates. Additionally, the degree of fear of COVID-19 is also an important risk factor for job preferences.

Studies have indicated that socioeconomic factors may have a profound impact on medical students’ career choices [8, 9, 23]. Our study showed significant differences in the proportions of current major, undergraduate years, and family members or relatives in healthcare in the single analysis. A comparatively higher proportion of students majoring in clinical medicine and traditional Chinese medicine exhibited a willingness to practice medicine in comparison to those who displayed unwillingness. Medical students are more likely to pursue a career in medicine after graduation than students in pharmacology. Studies have shown significant differences in professional abilities between clinical and non-clinical students, with clinical students displaying significantly higher levels of both intrinsic and extrinsic motivation than their non-clinical students [24]. Compared to the group without family members or relatives working in healthcare, the willingness rate in the group with family members or relatives working in healthcare was relatively higher to be willing to engage in the medical profession after graduation, which was inconsistent with a previous study conducted in Wuhan [25]. The different objective was the main reason. The study investigated the senior high school children of employees of a large hospital in Wuhan city [25], whereas we interviewed medical undergraduates in several of China’s higher medical schools. Our results indicated that family members or relatives working in healthcare had positive effects on recognition of their profession. During the COVID-19 epidemic, the psychological status of medical students may determine their career choice [20]. We used the GSES score to measure self-efficacy. The mean GSES score (2.83) in all participants was at the medium level, which indicated that medical students’ psychological resilience during the pandemic has remained strong, and they have not been adversely affected by it. The findings from this study showed that the self-efficacy of the medical students was lower in the unwillingness group, with a mean score of 2.73, compared with the willingness group. Meanwhile, in the multivariate analysis, we also found that a one-point increase in GSES score can increase the 87% proportion willing to engage in the medical profession as a career. Students with high self-efficacy were more steadfast in their career choices and had increased confidence to do the job well [26]. Therefore, the medical university could consider offering courses related to psychological well-being to enhance medical students’ self-efficacy, which can benefit their career choices.

We found several positive underlying motivations for career choice in this study. Reasons for choosing medicine majors in personal ideals, students who had family support, and students who thought of the high income and social respect in medicine were willing to engage in the medical profession as a career, which was consistent with others [27]. The attainment outcomes of students were predominantly attributed to the perceived enjoyment value of their academic pursuits. Medical careers are valued for the opportunity to have a positive impact on the lives of others, which can be a meaningful and fulfilling aspect of the profession. Students who did not self-select the medical major in the unwilling group accounted for 59.74% (463/775). This situation is likely because some medical students made their choice before they knew the specialty, resulting in a lack of interest in the learning processes, decreased career expectations, and reduced identification with the medical profession. Family support, mainly from parents, plays an important role in students’ career choice, which can provide a sense of emotional security and stability [28]. Medical students are confronted with more pressure and challenges. Family support can create a good family atmosphere for their children and avoid excessive or unrealistic expectations, reducing children’s pressure to some extent [29]. Income and social respect are important aspects of career needs or preferences [7]. Medical careers are often associated with high earning potential, making them an attractive option for those seeking financial stability and security. Medical professionals are highly regarded in society and often respected for their knowledge and expertise [30].

In this study, we found that tension in the doctor-patient relationship, heavy workload, and long training were the risk factors for undergraduates choosing medical work after graduation, which was consistent with previous studies [10, 14]. The extensive and intensive training required to become practice medicine in China can be both physically and mentally demanding, which may hamper some students from pursuing this. Additionally, the doctor-patient relationship can be quite stressful due to factors such as a large patient population, long working hours, and the high stakes involved in treating patients. These stressors can lead to burnout, job dissatisfaction, and even negative health outcomes among medical professionals. Therefore, to improve the rate of willingness to practice medicine for medical undergraduates, the government decision-maker should address these issues by improving the working environment, and alleviating the doctor-patient relationship. During the COVID-19 epidemic, we also found that the degree of fear of COVID-19 was an important risk factor for choosing the medical profession as a career. Compared with those who were very afraid of COVID-19 in this study, 73.96% of medical students who were not afraid at all about COVID-19 chose the medical profession. A study focused on career choice regret during the COVID-19 pandemic indicated that 5.3% of medical students made choice regret due to the outbreak of COVID-19 [31], which indicated that COVID-19 could affect career choice. The COVID-19 pandemic had negative impacts on the mental health of healthcare, increased workload and threatened to the safety of front-line medical workers, which increased levels of anxiety, depression, and stress [32, 33]. The weakening of the COVID-19 virus has reduced the risk to healthcare workers. Strengthening the correct education and promotion among medical students can help reduce their fear of COVID-19 and improve their willingness to practice medicine.

There are several limitations in our study. Firstly, this was a cross-sectional study, which could only evaluate career choice at a single moment without the longitudinal observation of the subjects. Therefore, a follow-up longitudinal study should be conducted to explore the changes in medical students’ career choices after they graduate. Secondly, we conducted the survey through an online platform, which may suffer from issues of selection bias. In our study, the sample had fewer freshmen and more low and high grade students. Finally, there may be other unmeasured confounding variables that could have influenced the results but were not accounted for in the survey. Further longitudinal studies are needed to address these limitations.

## Conclusions

The study highlights a noteworthy prevalence of medical undergraduates who expressed their willingness to pursue medicine as a career post-graduation. Several factors, including but not limited to current major, psychological factors, personal preferences, and career needs or preferences, were significantly associated with this willingness. Moreover, the impact of the COVID-19 pandemic on medical students’ career choices cannot be overlooked. Longitudinal studies can further explore the relationship between these factors and the willingness of medical students to engage in the medical profession in the future.

## Electronic supplementary material

Below is the link to the electronic supplementary material.

Supplementary Material 1

## Data Availability

The anonymized datasets used and/or analyzed during the current study are available from the corresponding author on reasonable request.
