# Supplementary material for: Willingness to practice medicine and related influential factors among medical undergraduates during COVID-19: a cross-sectional study
Source: BMC Med Educ. 2023 Jun 8;23:427. doi: 10.1186/s12909-023-04418-7 (PMC10249552; doi:10.1186/s12909-023-04418-7)
Supplement: Supplementary file 1 — Supplementary Material 1 [file 12909_2023_4418_MOESM1_ESM.docx]

***Supplementary***

Table S1 Variable codes for dependent and independent variables

| Variable | Code |
| --- | --- |
| Dependent | 0=unwillingness |
|  | 1=willingness |
| Current major | 0=Pharmacology |
|  | 1=Clinical medicine |
|  | 2=Dental medicine |
|  | 3=Nurse |
|  | 4=Traditional Chinese medicine |
| Undergraduate years | 1=Freshman |
|  | 2=Low grade |
|  | 3=High grade |
| Household income (thousands per year) | 1=≥201 RMB |
|  | 2=<50 RMB |
|  | 3=50~99 RMB |
|  | 4=100~200 RMB |
| Family members or relatives in healthcare | 0=No |
|  | 1=Yes |
| GSES score | Original values |
| Personal ideal | 0=No |
|  | 1=Yes |
| Family support for medical practice | 0=No |
|  | 1=Yes |
| High income | 0=No |
|  | 1=Yes |
| Social respect | 0=No |
|  | 1=Yes |
| Tension in doctor-patient relationship | 0=No |
|  | 1=Yes |
| Heavy workload | 0=No |
|  | 1=Yes |
| Long training | 0=No |
|  | 1=Yes |
| Afraid of COVID-19 degree | 1=Very afraid |
|  | 2=Afraid |
|  | 3=Not afraid |
|  | 4=Not afraid at all |
